# The Association between Medical Utilization and Chronic Obstructive Pulmonary Disease Severity: A Comparison of the 2007 and 2011 Guideline Staging Systems

**DOI:** 10.3390/healthcare10040721

**Published:** 2022-04-13

**Authors:** Chen-Yu Wang, Chen Liu, Hsien-Hui Yang, Pei-Ying Tseng, Jong-Yi Wang

**Affiliations:** 1 Department of Critical Care Medicine, Taichung Veterans General Hospital, Taichung 407752, Taiwan; chestmen@gmail.com; 2 Department of Nursing, Hungkuang University, Taichung 433304, Taiwan; 3 Respiratory Therapist, Asia University Hospital, Taichung 41354, Taiwan; janehp37@gmail.com; 4 Department of Academic Affairs, China Medical University, Taichung 406040, Taiwan; shyang@mail.cmu.edu.tw; 5 Department of Public Health, China Medical University, Taichung 406040, Taiwan; 6 Department of Medical, Lee’s General Hospital, Yuanli Town, Miaoli 358011, Taiwan; 7 Department of Health Services Administration, China Medical University, Taichung 406040, Taiwan

**Keywords:** chronic obstructive pulmonary disease, staging, medical cost, mortality

## Abstract

(1) Background: This study aimed to investigate the associations between the Global Initiative for Chronic Obstructive Lung Disease (GOLD) staging systems, medical costs, and mortality among patients with chronic obstructive lung disease (COPD). Predictions of the effectiveness of the two versions of the staging systems were also compared. (2) Purpose: this study investigated the associations between the Global Initiative for Chronic Obstructive Lung Disease (GOLD) staging systems, medical costs, and mortality among patients with COPD. Predicting effectiveness between the two versions of the staging systems was also compared. (3) Procedure: This study used a secondary clinical database of a medical center in central Taiwan to examine records between 2011 and 2017. A total of 613 patients with COPD were identified. The independent variables comprised the COPD GOLD Guideline staging of the 2007 and 2011 versions, demographic characteristics, health status, and physician seniority. The dependent variables included total medical cost, average length of hospital stay, and mortality. The statistical methods included binomial logistic regression and the general linear model (GLM). (4) Discussion: The total medical cost during the observation period for patients with COPD averaged TWD 292,455.6. The average length of hospital stay was 9.7 days. The mortality rate was 9.6%, compared with that of patients in Grade 1 of the 2007 version; patients in Grade 4 of the 2007 version had significantly higher odds of death (OR = 4.07, *p* = 0.02). The accuracy of mortality prediction for both the 2007 and 2011 versions of the staging was equal, at 90.4%. The adjusted GLM analysis revealed that patients in Group D of the 2011 version had a significantly longer length of hospital stay than those in Group A of the 2011 version (*p* = 0.04). No difference between the 2007 and 2011 versions was found regarding the total medical cost. Complications were significantly associated with the total medical cost and average length of hospital stay. (5) Conclusions: The COPD staging 2011 version was associated with an average length of hospital stay, whereas the COPD staging 2007 version was related to mortality risk. Therefore, the 2011 version can estimate the length of hospital stay. However, in predicting prognosis and mortality, the 2007 version is recommended.

## 1. Introduction

Chronic obstructive pulmonary disease (COPD) is currently the third leading cause of death, with a worldwide prevalence of 10.1% [1]. COPD was responsible for 3.2 million deaths in 2017 [2] and is projected to cause 4.4 million annual deaths by 2040 [3]. The proportion of patients with COPD has increased significantly in developing countries [1]. Yet, in Taiwan, COPD is still underdiagnosed, with an estimated prevalence of 6.1% [4]. The economic burden of COPD is high; in 2010, the estimated direct cost of COPD in the United States was USD 32 billion, and indirect costs were an additional USD 20.4 billion [5]. Further, the average annual cost of COPD in Taiwan prior to 2010 was TWD 3000 per person [6].

Since 2000, the diagnosis, staging, and management of COPD have followed the guidelines developed by The Global Initiative for Chronic Obstructive Lung Disease (GOLD), which is updated almost every year. The GOLD guidelines underwent extensive changes in staging in 2007 and 2011, respectively [7,8]. The basic components of COPD diagnosis include the symptoms, exposure history, and spirometry. COPD staging in the 2007 GOLD edition was primarily based on the degree of airflow limitation, whereas the 2011 edition also included symptom severity and exacerbation risk. In addition, different medications were recommended for each COPD stage in the 2011 edition.

Greater risk was observed with increasing COPD severity, regardless of the staging system. Patients with GOLD IV COPD had a 74.5% higher exacerbation rate, five-fold higher hospitalization rate, and three-fold higher intensive care unit (ICU) admission risk compared to those with GOLD I COPD [9]. The acute exacerbation and hospitalization rates of group D patients with COPD were close to three times that of group A patients [10]. Moreover, medical costs are presumed to increase according to the degree of COPD severity. In the literature, the medical cost was 2–3 times higher in stage III–IV patients than in stage I–II patients. Moreover, high-risk patients (Groups C and D) were associated with twice the medical cost compared to low-risk patients (Groups A and B) [11]. The cardinal medical cost comprises pharmacological expenditure, hospitalization, ICU admission, and emergency room (ER) visit [12,13,14,15,16].

A previous study investigated the health care resource utilization and medical costs associated with COPD using Taiwan’s National Health Insurance Research Database between 2004 and 2010. Because the study did not enroll patients with the 2011 COPD definition, spirometry was not available to confirm the COPD diagnosis [6]. Herein, we conducted a retrospective study to compare the health care resource utilization and medical costs associated with COPD according to the 2007 and 2011 COPD grading systems.

The aim of this study was to investigate the associations between the Global Initiative for Chronic Obstructive Lung Disease (GOLD) staging systems, medical costs, and mortality among patients with COPD. Predicting effectiveness between the two versions of the staging system was also undertaken.

## 2. Materials and Methods

### 2.1. Participants and Procedure

The inclusion criteria were COPD diagnosed by a pulmonologist, spirometry-proven obstructive lung disease, a history of COPD admission or ER visit, and regular follow-up at an outpatient department for more than one year. Patients with no spirometry data or who were less than 20 years of age were excluded. This retrospective study was approved by the Institute Review Board of the study hospital (IRB no: CE19031A). Informed consent was waived because of the retrospective nature of the obtained data.

### 2.2. Measures

Medical records between 2011 and 2017 were obtained from a tertiary medical center in central Taiwan and reviewed. After 2011, the GOLD guidelines included the Modified British Medical Research Council (mMRC) symptom assessment and the COPD Assessment Test (CAT) to assess severity of the symptoms. The symptom severity increases if the mMRC score exceeds 2 points or the CAT score exceeds 10. The collected data included COPD staging by 2007 and 2011 guidelines, age, sex, body mass index (BMI), smoking history, comorbidity, hypercapnia, and physician seniority. Outcome measurements included total medical cost, days of hospital stay, health care resource utilization, medical cost at end of life (EOL), and hospital mortality. Patients who consumed the top 10% of all medical costs in the preceding 12 months were considered to have high health care resource utilization [17]. Medical costs were calculated every three months for the year preceding EOL (EOL1, EOL2, EOL3, and EOL4) [18].

### 2.3. Statistical Analysis

The sample-size calculation of ordinal variables was based on a marginal error of 0.05 and an alpha level of 0.05. The assumed probability of two events occurring was 0.5. The minimum calculated sample size was 384. The sample size calculation of continuous variables was based on a confidence interval of 1.96, standard deviation of 7000, and desired marginal error of 600, requiring a minimum sample size of 523 to generate statistical significance.

Data were analyzed using the SPSS statistical software package (version 20.0; International Business Machines Corp., Armonk, NY, USA). Descriptive analysis of continuous variables, which are expressed as the mean and standard deviation, was conducted using the Mann–Whitney and Kruskal–Wallis tests. Differences in categorical variables were assessed using the chi-square and Fisher’s exact tests and are expressed as numbers and percentages. The association variables, medical cost, and days of hospital stay were analyzed with the general linear model. Independent variables include: (1) COPD staging, (2) demographic characteristics, (3) history of health, (4) physician experience. The dependent variables include: (1) total medical cost, (2) days of hospital stay, (3) High healthcare utilizers, (4) medical cost of every three months in EOL year, (5) mortality. A repeated measures analysis of variance (ANOVA) was used to analyze every seasonal medical cost one year prior to EOL. All tests were two-sided, and *p* < 0.05 was considered statistically significant.

## 3. Results

Among the 1627 patients screened, 613 were enrolled in the final analysis. According to the GOLD guideline staging, 176 (28.7%) patients were stage I, 271 (44.2%) were stage II, 142 (23.2%) were stage III, and 24 (3.9%) were stage IV according to the 2007 staging, while 19 (3.1%) were Group A, 378 (61.7%) were Group B, 18 (2.9%) were Group C, and 198 (32.3%) were Group D according to the 2011 staging. More than 50% of the patients were older than 80 years of age. Male was the dominant sex (93.1%). Cigarette exposure history was found in 501 (82.7%) patients and of these, 105 (17.3%) were current smokers. Cardiovascular disease (32.6%) and hypercapnia (40.3%) were the two major comorbidities (Table 1).

Table 2 shows that the average medical cost among patients (*n* = 431) was NTD 292,455.6 ± 347,557.3, and 10% of the patients were considered high utilizers of health care services. The average length of hospital stay was 9.7 days. The mortality rate was 9.6% (*n* = 613). EOL analysis was conducted on the 59 patients who died during the enrollment period. The high medical cost during the last three months of life, 0–3 months before EOL, was NTD 251,709.2 ± 239,787.5.

The general linear model (Table 3) revealed that after controlling for covariates, there was no significant association of the 2007 edition COPD staging with total medical cost (*p* = 0.860) and days of hospital stay (*p* = 0.069) (*n* = 319). In the 2011 staging edition, there was no statistically significant difference between the severity classification and total medical cost of patients with COPD (*p* = 0.838) (*n* = 319). However, the length of hospital stay differed among the four groups in the 2011 staging (*p* = 0.040).

Multiple logistic regression (Table 4) was used to analyze the relationship between COPD severity classification and high medical utilization (*n* = 319) and mortality risk (*n* = 603). Due to the small sample size, participants in Stage 1 and 2 according to the 2007 staging and Group A and B according to the 2017 staging were merged into the reference category, respectively. The logistic regression model revealed that the severity classification was not associated with high medical utilization.

Regarding the mortality risk, in the 2007 staging version, the results showed that Grade 4 had a higher risk of death, which was 4.067 times that of the reference group (*p* = 0.018). In the 2011 staging version, the results showed that there was no statistically significant association after controlling for covariates (Table 4).

**Figure 1:**
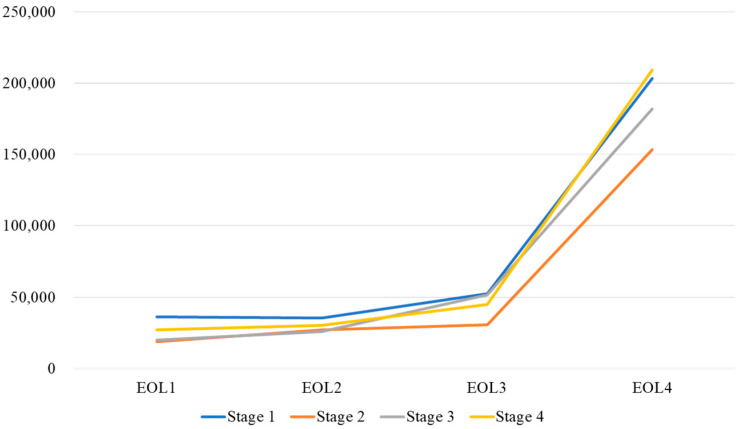
Medical cost in every quarter in the year before death in COPD patients using the 2007 COPD staging edition.

**Figure 2:**
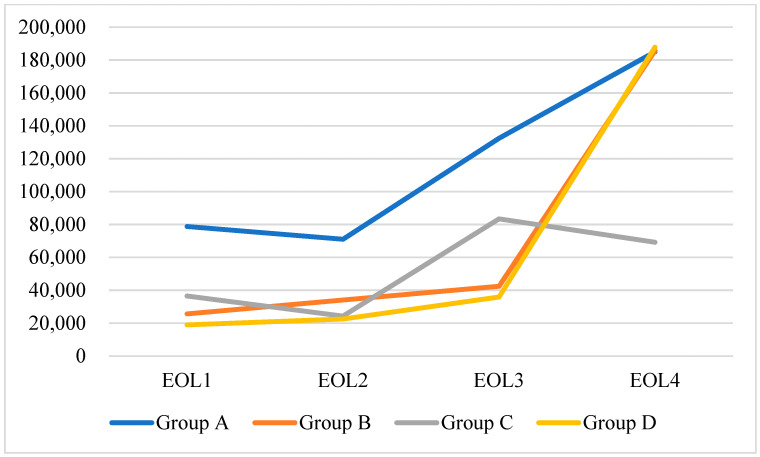
Medical cost in every quarter in the year before death in COPD patients using the 2011 COPD staging edition.

The last three months (EOL4) prior to EOL incurred higher medical costs than other periods (Table S1). There was a significant difference in COPD severity and total medical cost in the year preceding the EOL (Figure 1 and Figure 2).

## 4. Discussion

Chronic obstructive pulmonary disease in Taiwan ranks among the top ten in terms of medical costs, and the mortality rate is also high. Our study investigating health care utilization based on the severity of COPD found that the average medical cost per COPD patient was NTD 292,455.6. There was no difference in the medical cost among the four COPD stages in either staging scenario. Consistent with previous reports, the inpatient medical cost was the cardinal medical cost in more severe patients with COPD compared to those with less severe COPD [19]. In addition to disease severity, disease adherence and specialist-led care management were also found to be associated with medical cost in patients with COPD [7,8,20]. The Taiwanese healthcare system is characterized by brief outpatient visits, low costs, and a short waiting time [21]. The limited time of outpatient visits may explain the difficulty in delivering a complete care management plan for patients with COPD. Because Taiwan’s National Health Insurance system offers good medical care access, a short waiting time, low copayment, and lack of a referral system [21], the average number of hospital visits and drug prescriptions is significantly higher than in the member countries of the Organisation for Economic Co-operation and Development (OECD) [22]. Patients can visit a tertiary medical center for medication or admission regardless of their COPD severity, which may account for the lack of medical cost differences among patients with different degrees of COPD severity.

In our study, the length of hospital stay increased gradually according to the 2011 grading scale but not with the 2007 staging guidelines. In addition to spirometry, the 2011 guidelines included symptoms and annual exacerbation to rate the severity and guide the management of COPD. Acute exacerbation is a very important phenotype of COPD that is usually associated with frequent hospital admissions [23,24,25]. Group B patients showed more dyspnea symptoms and had higher mortality compared with Group C patients, who had better Fev1 results [26]. It is not surprising that symptomatic patients were associated with a longer hospital stay. However, most pooled data analyses showed that there was no difference between 2007 and 2011 regarding mortality prediction [27,28,29].

Notably, a recent study further integrated the dimension of ventilatory obstruction (FEV1) with the mMRC and CAT-based ABCD classification and found this new approach improved the ability to predict all-cause mortality [30]. Further study should determine whether this novel approach can improve prediction of medical utilization.

Among the patients studied, 43 were high utilizers of healthcare resources. Age, cardiovascular diseases, cancer, depression, and hypercapnia were also associated with high healthcare resource utilization. Severe COPD exacerbation has been reported as a cause of high healthcare resources utilization [31]. Although our study comprised a relatively small number of cases, the result was consistent with the risk factors for acute exacerbation previously reported [32,33].

Insight into advanced COPD is low compared to that of terminal lung cancer. Most patients with advanced COPD receive intensive care at the EOL [34]. A population-based study in the United States found that less than 15% of patients with advanced COPD receive palliative care [35]. In Taiwan, approximately 70% of do-not-resuscitate (DNR) decisions are made during the final hospital admission [36]. Delayed DNR decisions have been associated with high medical cost, including an increased ICU cost [37]. Our results were consistent with this finding among all stages of COPD in both staging groups, particularly in the last three months before the EOL. Early hospice intervention would help to reduce the medical cost of patients with COPD at the EOL. Interestingly, even patients with less severe COPD incurred higher medical costs in the three months preceding the EOL. A well-conducted study identifying the early risk factors for predicting EOL status in each COPD stage is needed [38].

There were four major strengths of this study. First, spirometry data were available from all enrolled patients. Second, the diagnosis was confirmed by a pulmonologist, making the diagnosis of COPD more reliable than that of previous large database studies. Third, we collected the detailed medical cost every three months before the EOL. Hence, we were able to pinpoint the medical cost in the last three months of life. Fourth, although this was a retrospective study, our sample size included 613 patients, which was higher than the estimated case number. However, there were also limitations in the study that must be acknowledged. First, this was a retrospective study; thus, a causal relationship was difficult to identify. Second, this study analyzed data from a single medical institute. The study participants were mainly older males. Consequently, the result’s generalizability is limited. Caution is needed when extrapolating the study results to other populations. Moreover, a multiple-center study is needed to extrapolate the current findings. Third, health care resource utilization calculation was based on the Taiwanese health insurance system. The results may not be generalizable to other countries without similar health care systems. Fourth, we probably underestimated the case number of patients with earlier stage COPD because less symptomatic patients may not seek medical help.

## 5. Conclusions

Because patients with COPD and multiple comorbidities were associated with higher medical costs and longer hospital stays, the COPD care plan should include associated comorbidities. The COPD staging 2011 version was associated with average length of hospital stay, whereas the COPD staging 2007 version was related to mortality risk. Therefore, the 2011 version can estimate length of hospital stay. However, in predicting prognosis and mortality, the 2007 version is recommended. This study provides information concerning the staging data of patients with COPD. The findings serve as a reference for medical institutions to prioritize high-risk COPD patients and allocate resources appropriately for preventive interventions and newer assessment systems. Future research could examine whether integrating the lung function dimension into the GOLD ABCD classification can improve the prediction of various adverse events and health care utilization.

## Figures and Tables

**Table 1:** Demographic data of COPD patients (*n* = 613).

Variables	*n*	%
**COPD staging 2007 Edition**		
Stage 1	176	28.7
Stage 2	271	44.2
Stage 3	142	23.2
Stage 4	24	3.9
**COPD staging 2011 Edition**		
Group A	19	3.1
Group B	378	61.7
Group C	18	2.9
Group D	198	32.3
**Age**		
20–59 (y/o)	41	6.7
60–79 (y/o)	262	42.7
≥80 (y/o)	310	50.6
**Gender**		
Female	42	6.9
Male	571	93.1
**BMI (kg/m^2^)** **(*n* = 508)**		
<18.0	26	5.1
18.0–20.9	78	15.4
21.0–23.9	153	30.1
24.0–26.9	138	27.2
≥27.0	113	22.2
**Occupation (*n* = 610)**		
Private and government employee	49	8.0
Farmer and fisherman	49	8.0
Labor union member	60	9.8
Soldier and veteran	113	18.5
Other	339	55.6
**Smoking History** **(*n* = 606)**		
Never	63	10.4
Smoking cessation	438	72.3
Current smoker	105	17.3
**Comorbidity**		
DM	63	10.3
Cardiovascular disease	200	32.6
Dementia	11	1.8
Cancer	101	16.5
Depression	6	1.0
Hypercapnia	247	40.3
**Physician experience (*n* = 330)**		
≤5 y	25	7.6
6–10 y	96	29.1
11–15 y	65	19.7
≥16 y	144	43.6

COPD, chronic obstructive pulmonary disease; BMI, body mass index; DM, diabetes mellitus.

**Table 2:** Medical cost, healthcare resource utilization, and mortality in COPD patients.

Variables	Mean/*n*	SD/%
**Total Medical Cost (NTD) (*n* = 431)**	292,455.6	±347,557.3
**Days of hospital stay (*n* = 431)**	9.7	±8.0
**High utilizers of healthcare (*n* = 431)**		
No	388	(90.0%)
Yes	43	(10.0%)
**Survival (*n* = 613)**		
Survival	554	(90.4%)
Death	59	(9.6%)
**Medical cost before EOL** **(*n* = 59)**		
10–12 months before EOL	61,750.1	±89,911.3
7–9 months before EOL	80,491.1	±116,641.9
4–6 months before EOL	104,740.1	±140,461.0
0–3 months before EOL	251,709.2	±239,787.5

COPD, chronic obstructive pulmonary disease; EOL, end of life.

**Table 3:** General linear model of total medical cost and days of hospital stay in COPD patients.

Variables	Total Medical Cost (*n* = 319)			Days of Hospital Stay (*n* = 319)		
	ln LS Mean ^1^	LS Mean ^2^	*p*-Value	ln LS Mean ^1^	LS Mean ^2^	*p*-Value
**COPD staging 2007 Edition**			0.860			0.069
Stage 1	12.719	334,034.647		2.254	9.526	
Stage 2	12.611	299,838.717		2.183	8.873	
Stage 3	12.680	321,258.059		2.349	10.475	
Stage 4	12.790	358,613.326		2.573	13.105	
**COPD staging 2011 Edition**			0.838			0.040 *
Group A	13.023	452,706.821		2.112	8.265	
Group B	12.661	315,211.777		2.237 ^a^	9.365	
Group C	12.737	340,101.710		2.526	12.503	
Group D	12.676	319,975.593		2.443 ^b^	11.508	
**Age**			0.009 *			0.573
20–59 y/o	13.447 ^a^	691,763.906		2.414	11.179	
60–79 y/o	12.281 ^b^	215,561.170		2.264	9.621	
≥80 y/o	12.370 ^b^	235,625.745		2.342	10.402	
**Gender**			0.585			0.850
Female	12.803	363,305.734		2.318	10.155	
Male	12.596	295,374.700		2.361	10.602	
**BMI (kg/m^2^)**			0.230			0.324
<18.0	12.408	244,751.821		2.439	11.462	
18.0–20.9	12.604	297,747.175		2.342	10.402	
21.0–23.9	12.762	348,711.426		2.352	10.507	
24.0–26.9	12.742	341,806.477		2.185	8.891	
≥27.0	12.982	434,521.194		2.381	10.816	
**Occupation**			0.260			0.625
Private and government employee	12.723	335,373.461		2.345	10.433	
Farmer and fisherman	12.837	375,870.519		2.319	10.166	
Labor union member	12.274	214,057.511		2.232	9.318	
Soldier and veteran	12.883	393,564.403		2.366	10.655	
Other	12.781	355,400.286		2.437	11.439	
**Smoking History**			0.720			0.783
Never	12.557	284,076.828		2.417	11.212	
Smoking cessation	12.774	352,921.171		2.312	10.095	
Still smoking	12.768	350,809.984		2.290	9.875	
**Comorbidity**						
DM	12.848	380,027.919	0.065	2.461	11.717	0.012 *
Cardiovascular disease	12.908	403,527.533	0.002 *	2.425	11.302	0.032 *
Dementia	12.849	380,408.137	0.414	2.377	10.773	0.737
Cancer	12.953	422,101.042	<0.001 *	2.291	9.885	0.225
Depression	12.907	403,124.207	0.411	2.522	12.453	0.230
Hypercapnia	13.059	469,301.172	<0.001 *	2.506	12.256	<0.001 *
**Physician Seniority**			0.116			0.854
≤5 y	12.285	216,425.142		2.255	9.535	
6–10 y	12.788	357,896.816		2.389	10.903	
11–15 y	12.843	378,132.522		2.354	10.528	
≥16 y	12.883	393,564.403		2.361	10.602	

COPD, chronic obstructive pulmonary disease; LS, least-squares; ln, natural logarithm. The covariates in the regression model included age, gender, BMI, occupation, smoking, comorbidity, and physician experience. An LS mean is significantly different from another LS mean if they have different superscripts (e.g., a and b). * *p*-value < 0.05. ^1^ We used the natural logarithm of total medical expenses and the average length of hospital stay to fit the normal distribution. ^2^ This LS mean is the number of antilogarithms extracted from the ln LS mean.

**Table 4:** Logistic regression model of high medical utilization and mortality stay in COPD patients.

Variables	High Utilizers (*n* = 319)		Death (*n* = 603)	
	Odds Ratio	*p*-Value	Odds Ratio	*p*-Value
**COPD staging 2007 Edition**				
Stage 1	(ref)		(ref)	
Stage 2	(ref)		0.916	0.804
Stage 3	2.138	0.106	1.114	0.789
Stage 4	1.025	0.978	4.067	0.018 *
**COPD staging 2011 Edition**				
Group A	(ref)		(ref)	
Group B	(ref)		0.888	0.892
Group C	0.723	0.459	1.824	0.576
Group D	1.722	0.498	1.059	0.949
**Age**				
20–59 y/o	(ref)		(ref)	
60–79 y/o	0.015	<0.001 *	2.280	0.444
≥80 y/o	0.030	<0.001 *	6.199	0.086
**Sex**				
Female	(ref)		(ref)	
Male	0.914	0.943	3.919	0.230
**BMI (kg/m^2^)**				
<18.0	(ref)			
18.0–20.9	0.507	0.814		
21.0–23.9	0.610	0.554		
24.0–26.9	1.509	0.625		
≥27.0	1.324	0.739		
**Occupation**				
Private and government employee	(ref)		(ref)	
Farmer and fisherman	0.331	0.269	0.640	0.548
Labor union member	0.041	0.035 *	0.490	0.368
Soldier and veteran	0.639	0.528	0.718	0.577
Other	0.309	0.072	0.874	0.801
**Smoking History**				
Never	(ref)		(ref)	
Smoking cessation	1.005	0.995	1.135	0.830
Still smoking	1.103	0.923	1.023	0.973
**Comorbidity**				
DM	0.856	0.755	2.640	0.008 *
Cardiovascular disease	3.678	0.012 *		
Dementia	0.303	0.419	5.859	0.008 *
Cancer	2.890	0.018 *		
Depression	23.339	0.007 *		
Hypercapnia	5.394	0.002 *		
**Physician Seniority**				
≤5 y	(ref)			
6–10 y	3.632	0.340		
11–15 y	3.409	0.365		
≥16 y	8.197	0.114		

The covariates in the regression model included age, gender, BMI, occupation, smoking, comorbidity, and physician experience. COPD, chronic obstructive pulmonary disease; BMI, body mass index. * *p*-value < 0.05.

## Data Availability

The datasets used in this study are available from the Ministry of Health and Welfare Taiwan, on reasonable request.

## Supplementary Materials

The following supporting information can be downloaded at: https://www.mdpi.com/article/10.3390/healthcare10040721/s1, Table S1: The medical cost among four periods prior to EOL in COPD patients.

## Abbreviation

Acronym	Full Text
BMI	Body Mass Index
COPD	Chronic Obstructive Lung Disease
DNR	Do-Not-Resuscitate
DM	Diabetes Mellitus
ER	Emergency Room
EOL	End of Life
GLM	General Linear Model
GOLD	Global Initiative for Chronic Obstructive Lung Disease
LS mean	least-squares means
ICU	Intensive Care Unit
NTD	Taiwan dollars: 1 USD = 28.3799 NTD
OECD	Organisation for Economic Co-operation and Development
OR	Operating Room
