# Supplementary material for: The Association between Medical Utilization and Chronic Obstructive Pulmonary Disease Severity: A Comparison of the 2007 and 2011 Guideline Staging Systems
Source: Healthcare (Basel). 2022 Apr 13;10(4):721. doi: 10.3390/healthcare10040721 (PMC9024555; doi:10.3390/healthcare10040721)
Supplement: Supplementary file 1 [file healthcare-10-00721-s001.zip › healthcare-1608271-supplementary.pdf]

Supplementary Materials

Table S1. The medical cost among four periods prior to EOL in COPD patients.

	EOL1	EOL2	EOL3	EOL4	*p*-value
**COPD staging 2007 Edition**					<0.001*
Stage 1	36,062.2	35,418.9	52,470.2	203,414.3	
Stage 2	18,920.5	27,200.8	30,668.8	153,276.7	
Stage 3	19,831.0	26,003.9	51,740.7	182,043.3	
Stage 4	27,119.3	30,394.0	44,981.2	209,190.4	
**COPD staging 2011 Edition**					
Group A	78,826.1	71,040.1	132,455.2	184,979.5	0.001*
Group B	25,668.0	34,098.2	42,489.0	185,720.9	
Group C	36,497.5	24,294.4	83,449.8	69,216.9	
Group D	18,977.3	22,561.5	35,918.2	187,775.1	
**Age**					<0.001*
20–59 y/o					
60–79 y/o	26,029.9	54,447.9	81,308.0	223,910.1	
≥80 y/o	23,482.2	23,765.7	33,928.1	166,541.5	
**Sex**					0.106
Female	75,811.1	111,971.5	248,202.4	341,123.5	
Male	23,600.0	28,738.7	41,150.9	177,726.3	
**BMI (kg/m^2)**					<0.001*
< 18.0	32,663.1	35,703.4	36,388.2	151,599.9	
18.0–20.9	11,395.8	21,332.8	13,069.0	99,012.4	
21.0–23.9	9907.0	13,319.7	59,754.5	212,989.9	
24.0–26.9	43,826.8	82,536.8	102,744.4	279,288.3	
≥ 27.0	60,355.1	39,222.3	51,072.4	225,483.0	
**Occupation**					<0.001*
Private and government employee	67,643.1	102,950.1	187,775.1	280,127.5	
Farmer and fisherman	8014.4	8518.5	15,459.8	167,543.8	
Labor union member	52,365.3	25,642.3	13,548.1	86,163.3	
Soldier and veteran	34,337.7	29,202.2	33,389.6	120,330.8	
Other	19,594.4	29,822.0	48,291.0	216,858.4	<0.001*
**Smoking History**					
Never	36,242.9	45,342.5	52,313.0	169,905.9	
Smoking cessation	23,600.0	31,257.0	49,168.1	187,399.9	
Still smoking	21,982.5	17,378.7	17,943.8	148,895.5	
**Comorbidity**					<0.001*
DM	29,822.0	41,647.6	46,073.8	156,216.8	<0.001*
Cardiovascular disease	23,248.6	30,121.7	50,919.4	159,213.3	<0.001*
Dementia	33,489.9	31,539.6	34,717.5	88,344.6	<0.001*
Cancer	17,500.8	28,310.8	68,665.4	201,591.8	0.001*
Depression	17,854.3	21,439.7	46,490.3	144,784.3	<0.001*
Hypercapnia	23,742.0	28,882.8	43,088.0	169,736.1	<0.001*
**Physician Seniority**					
≦5 yrs	15,321.3	16,932.7	31,008.0	89,054.2	
6–10 yrs	29,319.3	24,959.3	28,395.9	147,561.5	
11–15 yrs	17,396.1	33,322.9	99,409.2	182,773.0	

COPD, chronic obstructive pulmonary disease; BMI, body mass index; DM, diabetes mellitus; *p-value < 0.05
